# Enhanced production of bacterial cellulose with a mesh dispenser vessel-based bioreactor

**DOI:** 10.1007/s10570-024-06367-w

**Published:** 2025-01-29

**Authors:** Joshua Loh, Thora Arnardottir, Katie Gilmour, Meng Zhang, Martyn Dade-Robertson

**Affiliations:** 1 Living Construction Group, Hub for Biotechnology in the Built Environment, Department of Applied Sciences, University of Northumbria, Newcastle Upon Tyne, NE1 8 UK; 2 Living Construction Group, Hub for Biotechnology in the Built Environment, Department of Architecture and the Built Environment, University of Northumbria, Newcastle Upon Tyne, NE1 8 UK; 3 Living Construction Group, Hub for Biotechnology in the Built Environment, School of Architecture, Planning and Landscape, Newcastle University, Newcastle Upon Tyne, NE1 7RU UK

**Keywords:** Bacterial cellulose, Bioreactor, Static culture, Intermittent feeding, *Komagataeibacter xylinus*, Engineered living fabricator, Engineered living materials

## Abstract

Bacterial cellulose (BC) has gained significant interest for various applications due to its useful properties which include chemical stability and strong physical properties as well as its biocompatibility. However, conventional fabrication methods are limited by a lack of control over the form, particularly depth and thickness, restricting BC applications to 2D planar shapes. The production of BC is constrained by the formation of a pellicle at the liquid–air interface. To overcome this constraint and enable continuous pellicle formation, a method was established to combine intermittent batch feeding with a supportive mesh scaffold. Intermittent feeding supplies nutrients to the pellicle, promoting sustained formation at the liquid–air interface, while the supporting mesh anchors the initial pellicle to prevent submersion. This approach facilitates the continuous growth of BC pellicle at a controlled rate. Moreover, the method described here results in a single BC pellicle with enhanced thickness, weight (both wet and dry states), water-holding capacity and mechanical strength. Continuous growth is achievable with ongoing nutrient supply, allowing for precise control over the final pellicle thickness. This culturing method is scalable and has been tested for volumes ranging from 250 mL to 10 L, and significantly increases glucose-to-cellulose conversion (3.4-fold) and water utilization efficiency (2.4-fold).

**Supplementary Information:**

The online version contains supplementary material available at 10.1007/s10570-024-06367-w.

## Introduction

Cellulose is the most abundant organic polymer on earth, and it is a linear polysaccharide comprising units of β-d-glucopyranose that are interconnected by β-1,4 glycosidic bonds (Klemm et al. 2005). While it is a basic structural material for plants, certain types of bacteria, such as *Komagataeibacter*, also produce cellulose, i.e. bacterial cellulose (BC). Unlike cellulose derived from plants, BC is high in purity and crystallinity and a different structural organisation to plant cellulose, and as such, has different material properties and characteristics from plant-based cellulose (Klemm et al. 2005; Czaja et al. 2006; Cheng et al. 2009). These characteristics lead to properties such as high mechanical strength, high water holding capacity, porosity, biodegradability, high stability, and biocompatibility. With further modification through biological, chemical, and/or physical approaches (Cheng et al. 2009; Florea et al. 2016; Hur et al. 2020; Gilbert et al. 2021; Gilmour et al. 2023), BC materials have found promising applications in the field of biomedicine (Czaja et al. 2006), electronics (Zeng et al. 2014), biosensors (Lv et al. 2018), energy storage (Zhang et al. 2021), insulation (Wang et al. 2023) packaging (Padrão et al. 2016) and textiles (Kamiński et al. 2020).

When grown statically, new layers of cellulose form at the air to liquid interface. BC production peaks after 5 to 7 days, after which cell growth and BC production reach a point of stagnation (Chao et al. 1997; Klemm et al. 2001; Hsieh et al. 2016). It is thought that production stagnation occurs because the BC pellicle acts as a diffusion barrier, which limits the supply of substrates and nutrients to the most active region for BC production (Klemm et al. 2001; Hornung et al. 2006; Lin et al. 2014; Hsieh et al. 2016). For continual BC production, this region requires the appropriate amount of O_2_ and supply of nutrient and substrate. High oxygen tension is crucial for the bacteria to produce BC, and it has been demonstrated that nascent BC production occurs at the surface layer and subsequent production leads to the submergence of latter layers (Klemm et al. 2001; Hsieh et al. 2016). Thus, the thicker the BC pellicle, the greater the barrier for diffusion becomes leading to stagnant production of the BC pellicle.

To overcome stagnation in BC production, various solutions have been explored, including reduction of friction forces (Hornung et al. 2006; Żywicka et al. 2018), use of additives (Ho Jin et al. 2019; Sani & Dahman 2010), agitation (Chao et al. 1997; Krystynowicz et al. 2002), and direct feeding (Hornung et al. 2007; Hsieh et al. 2016). Reducing the friction of the growth vessel associated with BC production has been shown to increase thickness in BC production by diminishing the binding force of the pellicle on the vessel walls, thus enhancing nutrient diffusion to the air–liquid interface (Hornung et al. 2006; Żywicka et al. 2018). In addition, various modes of agitation have been explored to improve nutrient and oxygen distribution during BC production, and they have been implemented into two different types of bioreactors: rotating and airlift. The first type employs the usage of rotating perforated discs/drums (Krystynowicz et al. 2002). The constant and slow rotation (4 rpm) ensures even distribution of nutrient, substates, and oxygen over the body of the discs/drums. This cyclical submersion into the medium and exposure to air encourages BC pellicle formation over the porous discs/drums. It has been observed that this mode of agitation can lead to continuous BC formation, resulting in even thicker BC production over time. As such, rotating reactors are advantageous for producing bulk cellulose. However, BC produced in such a manner may not make efficient use of nutrients and lack form control relevant for different applications. This method, while effective for the intended application, necessitates the manual intervention in extracting the BC from the discs and may result in material stress that potentially compromises the integrity of the cellulose material structure, leading to tearing in the material. In contrast, airlift bioreactors have been shown to be capable of producing BC by injecting a constant stream of gas from the bottom of the vessel (Krystynowicz et al. 2002). The injected air is designed to both aerate and agitate the culture within the bioreactor (Chao et al. 1997; Cheng et al. 2002; Song et al. 2009). It was observed that when the reactor was supplied with fortified O_2_, BC cultures can reach a higher cell density phase sooner and form a higher mass of BC (Chao et al. 1997). While aeration is capable of increasing BC productivity, it also produces BC that is lacking in form and shape resulting in uneven distribution of material and lack of control in the formation of BC (Cheng et al. 2002). For many applications, control over the form of the material is important as it significantly influences its mechanical performance and, consequently, its suitability for various purposes. In addition, some studies reported that agitation can lead to Cel (-) phenotype, in which the cellulose-producing bacteria mutates and loses the ability to produce BC pellicles due to the genetic instability brought about by over aeration (Hur et al. 2020).

Another strategy to overcome BC production stagnation is to employ batch feeding or direct feeding. Through direct feeding nutrients can be delivered to the air–liquid interface to enhance BC production. Hornung et al. (2007) developed a special bioreactor in which aerosolised growth medium is utilized to supply nutrients directly to the top of the BC pellicle and enable the continuous production of BC. Direct feeding has the advantage of indefinite growth and controllability of the speed and extent of growth of BC on the Z-axis. Challenges have arisen in generating aerosolized medium and effectively harvesting the BC pellicle due to contamination issues, as emphasized by Hornung et al. (2007), impacting BC material quality and integrity. Despite these challenges, direct feeding, as discussed by Hsieh et al. (2016), offers the potential benefit of influencing the final form of the BC material. However, it introduces disadvantages like delamination and regions of weakness within the BC structure, increasingly noticeable with a higher volume of medium between growth intervals. Mitigating this issue involves using a lower medium volume (Hsieh et al. 2016), emphasizing the importance of a careful balance in medium volume to prevent delamination.

For various potential applications of BC, it is critical for BC to be produced as a bulk material and to exhibit a three-dimensional form. This paper presents a novel bioreactor design that utilises intermittent batch feeding coupled with a mesh scaffold to continuously produce BC. At the small-scale test (< 1L capacity), the BC pellicles produced were thicker than conventional methods and could theoretically grow indefinitely. The production of BC pellicle was then upscaled to a 10 L capacity bioreactor and produced a pellicle that is over 80 mm in thickness. The mechanical properties of pellicles produced by this method showed an increase in Young’s modulus and tensile strength. The outcomes of this innovative biofabrication technique illustrate its scalability and enhanced utilisation of water and nutrients, offering potential applicability for industrial-scale BC production.

## Materials and methods

### Design and fabrication mesh dispenser vessel (MDV) based bioreactor

The design concept for the MDV Based Bioreactor comes from the need to control and predict the cellulose formation to enable the selective modification of material properties in the BC based materials as it is grown. This system is part of a BC fabrication system we refer to as Engineered Living Fabricator (ELF) (Dade-Robertson et al. 2023). The ELF is a system developed for the project *Living Manufacture* and is based on a liquid handling robot (Faina et al. 2020), is designed to support the guided growth of BC optimising the speed and thickness of BC pellicle production, enabling the modification of the pellicles through the addition of chemical and biological modifiers. These modifiers can be applied to the surface of the growing pellicle to achieve variable properties throughout the BC material.

A key challenge to the development of ELF system is achieving controllable growth of BC pellicles in terms of speed and consistency, as well as thick volumes. We proposed that such a system could be achieved if a vessel were developed in which the pellicle is initially grown over the mesh scaffold and nutrients are continually added. This process would mean that the pellicle is anchored to the mesh and BC is produced continually at the liquid–air interface, building BC volume as the liquid level rises.

**Fig. 1:**
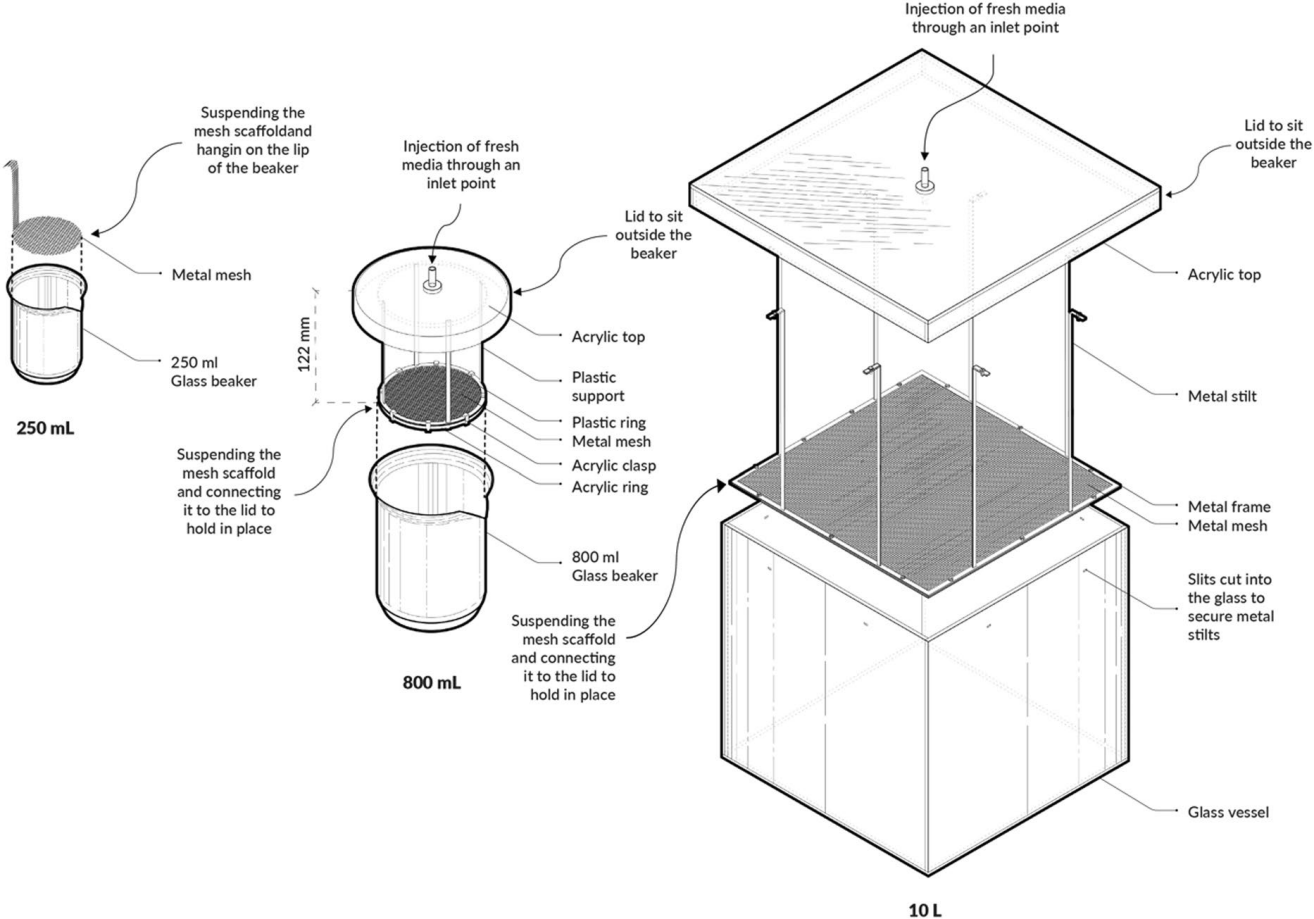
Design diagrams of the mesh dispenser vessel (MDV) Based Bioreactor prototypes in the three scales

The supportive mesh scaffold was initially developed and explored in small volume cultures (250 mL and 800 mL) before being scaled up for the ELF system at a 10 L scale (Fig. 1). The initial versions, with a capacity of 250 mL, underwent multiple iterations for rapid optimisation of the feeding strategy and selection of the optimal mesh aperture size. Initially, the supportive scaffold consisted of a single piece of mesh hooked to the lip of the beaker. This supportive mesh functions as an anchor for the initial pellicle formation to adhere to the mesh as growth media is introduced from the top of the vessel. This design prevents submersion of the BC pellicle as it gains weight and enables easy extraction of the pellicle. Through this scale, the correct size of mesh was selected. The 800 mL version was refined by ensuring the mesh was levelled at the liquid–air interface and incorporated a lid with a central tube attachment to minimise BC formation evenly cross the surface area. Growth medium was then introduced into the vessel from above using peristaltic pumps. Additionally, the lid also suspended the mesh scaffold in place and facilitated the introduction of fresh growth medium directly from the top of the vessel.

In scaling up the bioreactor vessel for the ELF system, a custom-cut 10 L glass container was utilised. The glass vessel itself featured inserts to anchor the mesh scaffold, separating it from the lid, and included a central point for media injection from above. The mesh could then be lifted out separately from the lid opening to preserve the intact pellicle for post- production processing.

**Fig. 2:**
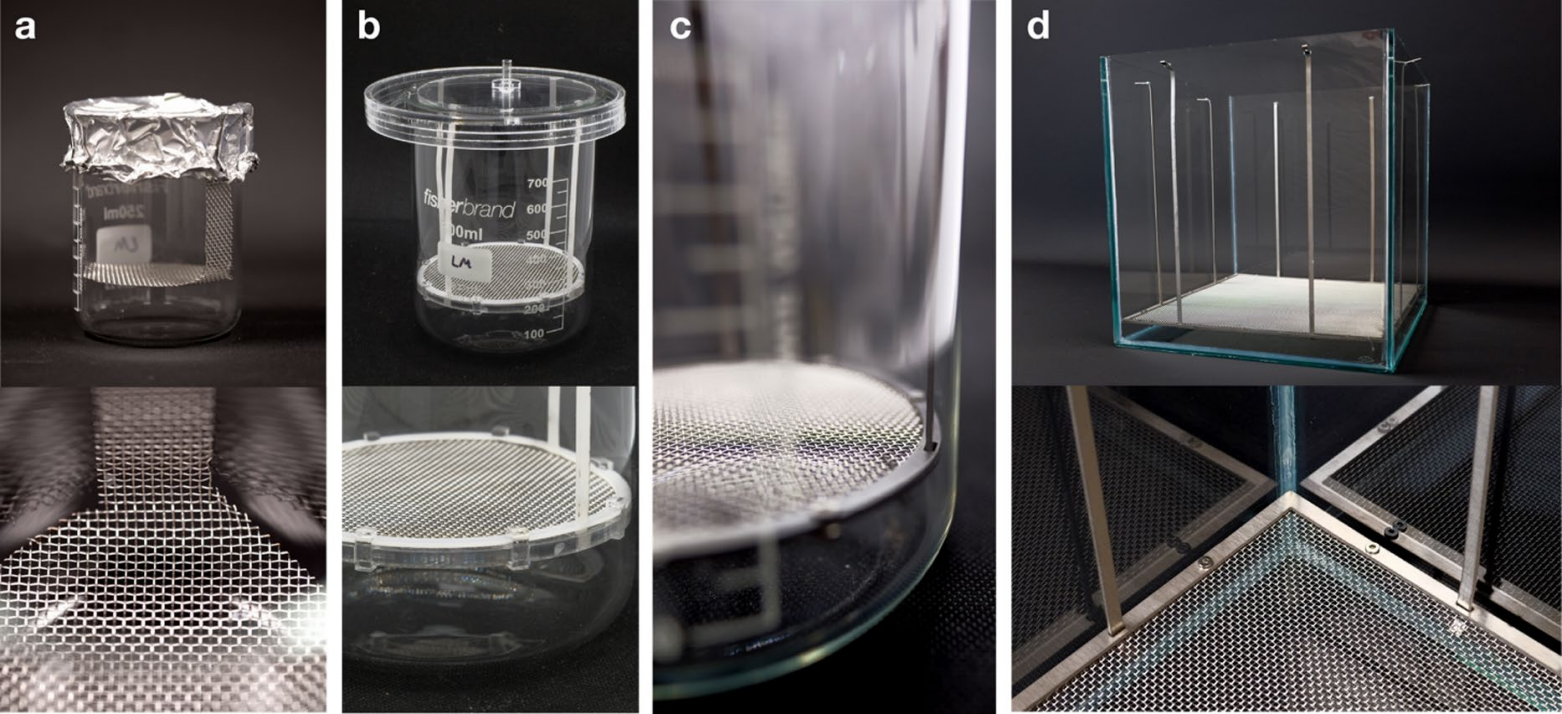
Photographs for different size of bioreactors. **a** 250 mL setup; **b** 800 mL setup with mesh in plastic; **c** 800 mL setup with mesh in metal, and **d** the 10 L glass setup

Each part of the MDV (Fig. 1), including the 10 L glass vessel, lids, frames, and stilts were designed in Rhinoceros 3D (version 7, McNeel, USA) and the files exported for fabrication. The vessel was fabricated from clear 4 mm acrylic sheets and 1 mm Acetal plastic sheets for the first 800 mL prototype (Fig. 2b), were laser cut by Cad Cam Technology FB 7100. Stainless steel sheets with a thickness of 1 mm were used to fabricate the mesh scaffold form of the 10 L prototype. Both the sheets and the mesh were precisely cut to specification using the ProtoMAX Waterjet cutter from 304 stainless steel woven mesh, featuring an aperture size of 1 mm and a thickness of 1 mm. The mesh was then fixed onto the frame by two metal frames and screwed into place with stainless steel nuts and bolts. The stilts were then placed into slits that were situated on the steel frame and lid (for the 800 mL), and in the glass slits (for the 10 L). These were fixed into place by bending the ends of the stilts to form hooks. To further maintain sterility, every part of the bioreactor was designed for autoclave or chemical sterilisation.

When BC was produced in 250 mL size beakers, initially three mesh aperture sizes (0.18 mm, 1.0 mm, and 2.0 mm) were used to test pellicle attachment to the supporting mesh. The meshes were cut into a circular supporting base with a diameter of 60 mm and 10 mm width strips on one side to be suspended on the lip of the beaker. The mesh scaffold support was suspended to the height of 2.5 cm (equivalent to the volume of 80 mL) above the base of the beaker. This beaker set up was sealed with aluminium foil and was then sterilised by autoclave. The dimensions of each of the parts for the 800 mL setup are a diameter of 95 mm for the mesh steel frame, a diameter of 140 mm for the lid, and a height of 107 mm for the length of the stilts. The total height of the lid to mesh is 122 mm. For the 10 L glass vessel with dimensions of 250 × 258 mm, the mesh steel frame had a diameter of 248 mm × 240 mm, the lid had a diameter of 290 mm × 298 mm, and stilts with a height of 220 mm were fabricated. The total height from the lid to the mesh was 238 mm.

### Bacterial and culture conditions

In all experiments *Komagataeibacter xylinus* DSM 2325 was used as the cellulose-producing bacterium. *K. xylinus* was cultured in Hestrin-Schramm (HS) medium, which consisted of glucose (20 gL^−1^), peptone (5 gL^−1^), yeast extract (5 gL^−1^), citric acid (1.15 gL^−1^), and disodium phosphate (2.7 gL^−1^). The HS medium was then titred to pH 6.0. *K. xylinus* cultures were initially grown in 10 mL of HS medium for 3 to 5 days. Once a pellicle was formed, it was treated with 1% (v/v) cellulase from *Trichoderma reesei* (Sigma-Aldrich, C2730) at 28 °C and 200 rpm until the BC pellicle was completely lysed. To remove the cellulase, cells were then pelleted by centrifugation and washed with HS medium 3 times before being resuspended in the same volume of HS medium.

### BC production with 250 mL beaker

A 250 mL beaker and a mesh support were sterilised by autoclaving. Sterile HS medium was added to the beaker set up to achieve a volume of 80 mL. The initial medium was to establish the meniscus at the height of the mesh scaffold support. *K.* x*ylinus* starter culture was used to achieve 1% v/v of inoculum. Over the course of 28 days at 28℃, 6 mL of HS medium were delivered per day for single feeding to achieve 1 mm of coverage over the BC pellicle. The thickness of the pellicles was measured with a digital caliper (Mitutoyo Absolute Digimatic) daily. All cultures were produced in triplicate.

### BC production with 800 mL beaker

For the culture a 800 mL beaker, a beaker was sterilised by autoclaving, and the plastic version of the mesh apparatus (Fig. 2a) were sterilised under a UV lamp for 30 min. Once sterile, 200 mL of HS medium was added into the beaker which forms a meniscus on the mesh. All production cultures were then incubated at 28 ℃ and under static conditions with mesh support. During culturing, 9 mL of HS medium was added daily to the culture to achieve an increased medium height of 1 mm above the pellicle to a total volume of 400 mL. All cultures were inoculated with 1% (v/v) of *K. xylinus* starter culture. The thickness of the pellicles was measured with a digital calliper daily. Controls were set up as described above without mesh. All cultures were produced in triplicate.

### BC production with 10 L glass vessel

In the scale up experiments, a 10 L glass vessel was used. The glass vessel and scaffold apparatus were sterilised by autoclaving. The lids to the bioreactor were sterilised with 70% EtOH. When setting up the cultures, 1.2 L of HS medium was added to the bioreactor and additional HS medium was added to form the meniscus of the medium to sit at the surface of the mesh. Cultures were then inoculated with 1% (v/v) of *K. xylinus* starter culture. Intermittent medium feeding delivery was achieved with automated peristaltic pumps (Fisher scientific, Microflex™ Compact Single-Channel Pump, UK), which delivered sterile HS medium at a rate of 70 mL per day and is the equivalent of 1 mm of height per day. The thickness of the pellicles was measured with a digital calliper. In total, 3 BC pellicles were produced with a cultivation time of 48, 60, and 96 days.

### Post-production processing

BC pellicles produced from 250 and 800 mL cultures were harvested at the end of experiment. Pellicles were removed from the vessel and peeled from the supporting mesh followed by washing with sterile distilled water to remove excess medium. The pellicles were then washed in 0.1 M NaOH at 90 °C for 30 min to remove bacterial cells and media components and were subsequently washed with sterile distilled water. The wet weight of the pellicle was measured once excess water was no longer produced from the washed pellicle. BC pellicles were then dried by freezing in -80 °C overnight and lyophilised for 2 to 3 days. The dry weight of the pellicle was then measured.

BC pellicles produced from 10 L cultures were harvested at the end of experiment. Pellicles were removed from the vessel and peeled from the supporting mesh followed by washing for 16 h with sterile distilled water. The pellicles were then washed in 1 M NaOH with equal volume for 16 h and followed by 6 h wash with sterile distilled water. The BC pellicle was dried in a constant environment chamber (Memmert Constant Climate Chamber) set to 35 to 40 ℃ and 15% relative humidity. During drying, the pellicles were placed between a metal mesh and steel plate to ensure the BC dried evenly.

The dried pellicles were then weighed to obtain the yield of cellulose, and the thickness of the dried pellicles was recorded. From this data, the dry density could be calculated based on the dry weight over the volume of the samples.

#### Morphological analysis of BC with SEM

Dried BC pellicle was coated with 4 nm Pt or Cr using a sputter coater (Quorum Q150V ES). Micrographs of pellicles were obtained from TESCAN MIRA3.

### Mechanical properties

Upscaled cultures produced BC pellicles that were cut with either a bandsaw or scalpel, depending on their thickness, into five strips according to measurements (20 cm × 4 cm) dictated by ASTM D883. The mass and volume of each strip was then recorded, and the tensile strength of this material was measured according to the standard protocol using Instron (Instron 68TM-50 with Bluehill Universal Version 4.21 software) to obtain tensile stress / strain curves from which the Young’s modulus could be determined.

### Glucose assay with HPLC

1 $$\text{\%Volume usage}=\frac{\text{Pellicle volume}}{\text{Total medium volume}}$$

%Volume usage derived from the volume of media used in the bioreactor.

2 $$\text{\%}Glucose conversion=\frac{\text{Total pellicle dry weight (g)}}{\text{Total glucose added (g) - Glucose remaining (g)}}$$

%Glucose Conversion derived from the yield of BC production over the total of glucose present in the system.

To measure the amount of glucose in the culture, an aliquot of 1 mL was removed from the BC production culture at the beginning and end of culturing. The medium was then frozen until further processing. Samples are then run on a HPLC, Dionex ICS-5000 + DC with a Dionex CarboPac PA200 column (3 × 250 mm) with a program that runs for 50 min with a linear gradient containing buffer A (100 mM NaOH) and buffer B (100 mM NaOH and 500 mM NaOAc) with solvent B from 0 to 60% in 50 min and solvent B held at 100% for a further 10 min. The flow rate is set to 0.5 mL per min and with an injection volume of 200 µL. A stock solution of glucose was prepared with HS medium. A series of glucose-HS medium were made to the concentrations of 20 gL^−1^, 2 gL^−1^, 0.2 gL^−1^, 0.02 gL^−1^, and 0.002 gL^−1^ to construct the calibration curve. The data was analysed with Chromeleon 6.0, and glucose peaks were quantified by calculating the area under the curve. The calculation for volume usage and glucose conversion are defined in Eq. 1 and 2 respectively.

## Results and discussion

In this study, we developed and optimized a novel bioreactor for BC production. We investigated the impact of a supporting mesh scaffold and intermittent feeding in this MDV based system to address the challenge of BC stagnation, often caused by limited nutrient distribution to the top of the pellicle. By implementing intermittent feeding, we demonstrated that BC pellicles could surpass the stagnation threshold, enabling indefinite and continuous BC production while yielding materials with advanced physical and mechanical properties.

### Optimisation of mesh aperture for MDV

**Fig. 3:**
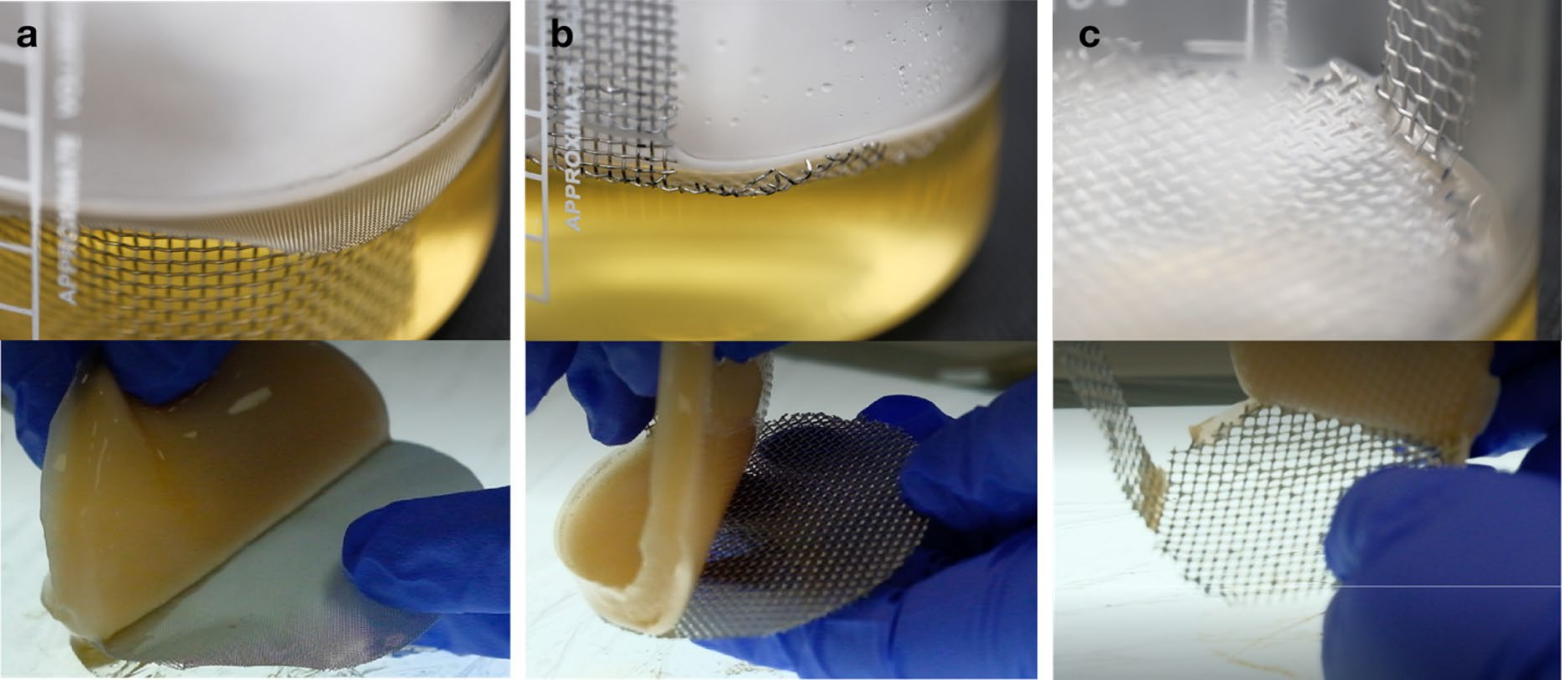
The impact of mesh aperture on adhesion on the BC pellicle. The mesh was made from stainless steel and cut to sizes for supporting BC production and with the following aperture size: **a** 0.18 mm, **b** 1 mm, **c** 2 mm

Initially, the investigation focused on testing the impact of mesh aperture size on BC production, particularly assessing its effect on the ease of harvest (Fig. 3). At the smallest aperture (0.18 mm), there was no adhesion between pellicle and mesh. With this configuration, the pellicle was physically supported solely by the mesh and did not have any immobilisation effect on the BC pellicle. The lack of immobilisation may result in unwanted pellicle agitation due to the addition of growth medium. At larger aperture sizes (1.00 mm and 2.00 mm), the pellicle was integrated into the mesh structure, resulting in the anchoring of the pellicle. However, at 2.00 mm, the pellicle was integrated to a greater degree than that observed at the 1.00 mm aperture. Due to the greater integration, harvesting the pellicle was more difficult, resulting in greater loss of BC pellicle during the harvesting process. At a 1 mm aperture, it was easier to extract the pellicle from the mesh while minimising loss of material. Since the mesh was positioned at the meniscus, the pellicles that were able to integrate with the supporting mesh resulted in the immobilisation of the formed pellicle. With these observations in mind, a mesh aperture size of 1.00 mm aperture size was used for further testing.

### Effects of intermittent feeding and mesh support scaffold on BC production

**Fig. 4:**
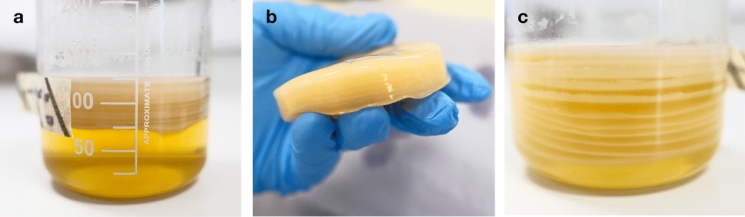
Effects of supporting mesh scaffold and intermittent feeding on BC pellicle production. **a** BC pellicle produced with a supporting mesh and intermittent feeding; **b** harvested pellicles produced from a culture subject to supporting mesh scaffold and intermittent feeding; **c** BC pellicle produced through intermittent feeding without the mesh

When intermittent feeding and supporting mesh was used during the formation of BC, the cultures produced BC that were thicker and did not laminate (Fig. 4a). The mesh provides a support that prevents the submersion of the initial BC pellicle. During the initial phase of culturing, nascent pellicles were formed on the meniscus of the medium. The addition of medium at a daily interval led to further BC formation and resulted in an increased pellicle thickness. Following Hsieh et al. (2016), a daily intermittent feeding rate of 1 mm of total medium in depth was explored. The addition of fresh medium provides nutrients for further pellicle formation. In comparison to the control, the addition of the medium without the mesh can result in the submersion of BC pellicle and the formation of multiple layers, as it can be seen in Fig. 4c. While the supporting mesh prevents submersion of pellicles to create a thicker pellicle (Fig. 4b).

Control cultures that were subjected to intermittent feeding and did not have a supporting mesh scaffold produced a total of 13 discrete layers of pellicle within 31 days (Fig. 4c) that did not structurally adhere to each other. The formation of multiple layers was attributed to the addition of growth medium along with the increased weight of the top pellicle layer. When a BC pellicle is formed, the addition of fresh medium submerges the topmost pellicle. Over multiple feeding events, this contributed to the formation of multiple discrete layers. The observation of multiple layers of pellicle was also described in studies that have utilised intermittent feeding (Hsieh et al. 2016). In their work, it was also observed that by changing the feed volume and feed interval, the layering effect can be controlled (Hsieh et al. 2016). With the intermittent feeding method, the barrier for diffusing nutrients becomes a non-factor, as nutrients are delivered directly to the active growing zone rather than nutrients supplied via diffusion from beneath the pellicle in most conventional static cultures without mesh support. In this work, it was found that a daily feed rate to achieve approximately 1 mm of medium coverage in height over the formed pellicle was optimal. At 1 mm per day feed rate, in conjunction with a mesh support, the laminations are minimised and pellicle layer formations are tightly bonded together after harvesting.

### Producing BC pellicles with a supporting mesh scaffold and intermittent feeding at 800 mL scale

Next, pellicles were produced with a more refined design of the supporting mesh scaffold and incorporated findings from the previous optimisation stage. This setup included the use of 1 mm mesh aperture and utilises a peristaltic pump to enable a feed rate that achieves a 1 mm height of fresh medium. With MDV1, pellicles were produced at 800 mL scale and cultured for 28 days.

**Fig. 5:**
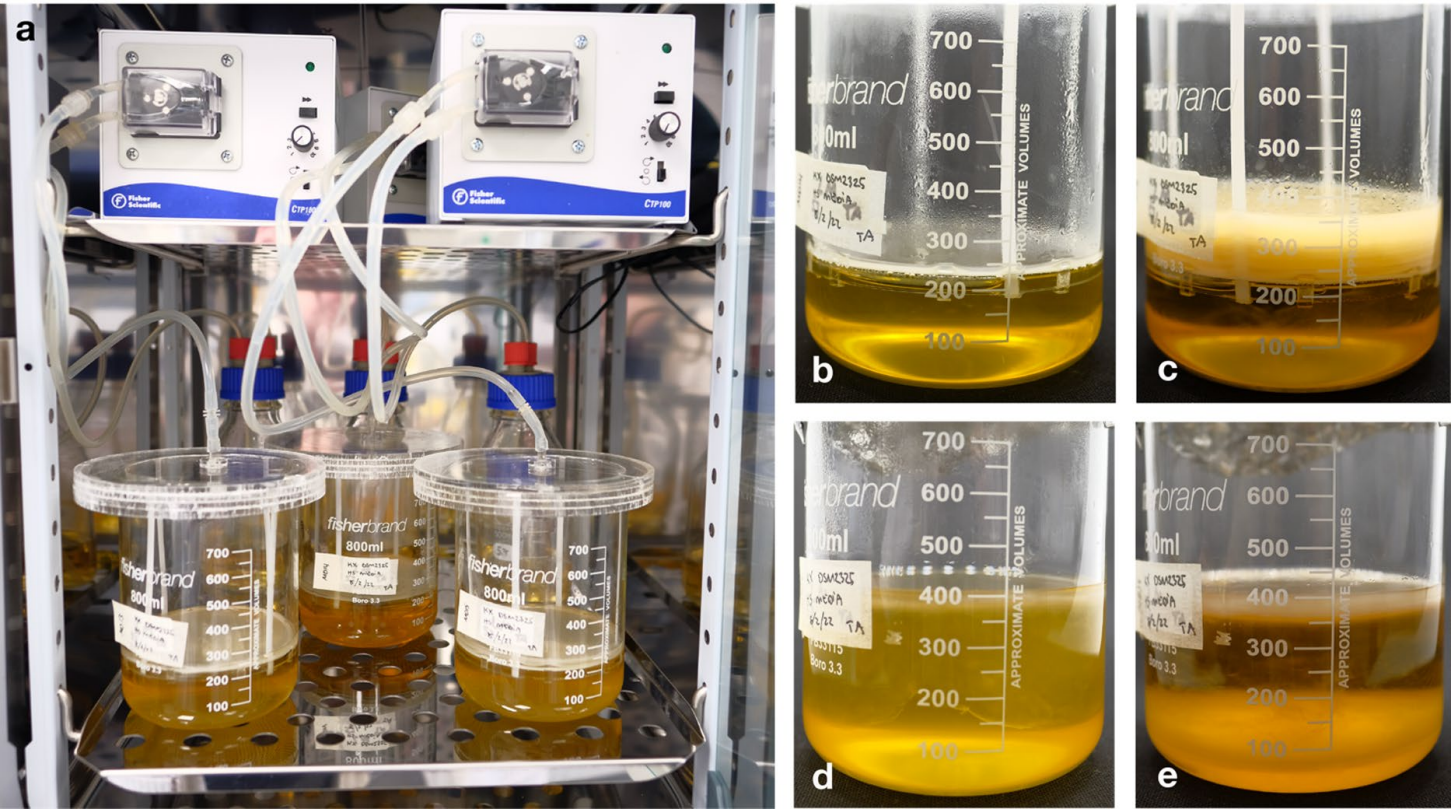
BC formation in 800 mL MDV1 based bioreactors. **a** Intermittent feeding setup of the 800 mL scale; **b** Intermittent feeding, with mesh, of BC with mesh after 4 days of culturing; **c** Intermittent feeding, with mesh, of BC after 28 days of culturing; **d** Conventional static BC without mesh after 4 days of culturing; **e** Conventional static BC without mesh after 28 days of culturing

**Fig. 6:**
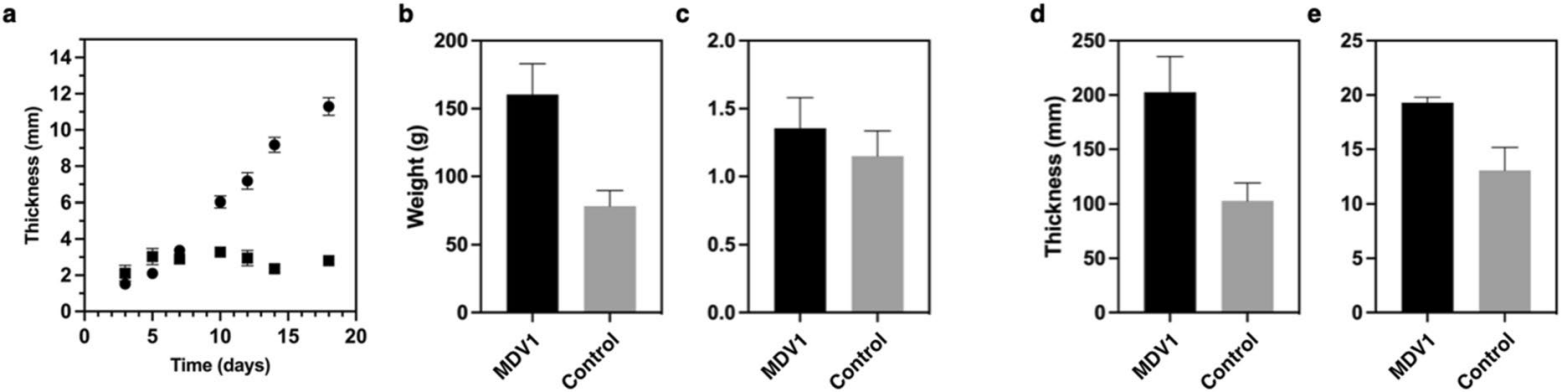
Physical characteristics of MDV cultures in 800 mL scale. **a** Thickness of BC pellicle during culturing with MDV (circles) against control (squares); **b** wet weight **c** dry weight **d** wet thickness and **e** dry thickness. Error bars indicate standard error of mean and n = 3

BC pellicles produced from these cultures were similar to those observed in 250 mL cultures, where the pellicles grew thicker over time as the medium was topped up (Fig. 5b, c). Similarly, pellicles produced at this scale did not delaminate during the harvesting and post-production processing. When observing both cultures over the first 4 days, the rate of production for BC (in terms of thickness) was similar. This suggests that the method described here does not improve the rate of BC production at the initial stage. When comparing static conventional cultures without mesh to pellicles produced with a supportive mesh and intermittent feeding, the physical characteristics were observed to be superior. The characteristics observed include lengths, wet weight, dry weight, wet thickness, and dry thickness (Fig. 6b-e). Results here suggest that the pellicles produced with a supportive mesh and intermittent feeding also have a higher water holding capacity. This increase in water holding capacity is likely due to a looser network of cellulose forming between layers during intermittent feeding, thus allowing a larger volume of more porous BC and resulting in a greater water holding capacity.

### Upscaling BC production with mesh and intermittent feeding to 10 L scale

**Fig. 7:**
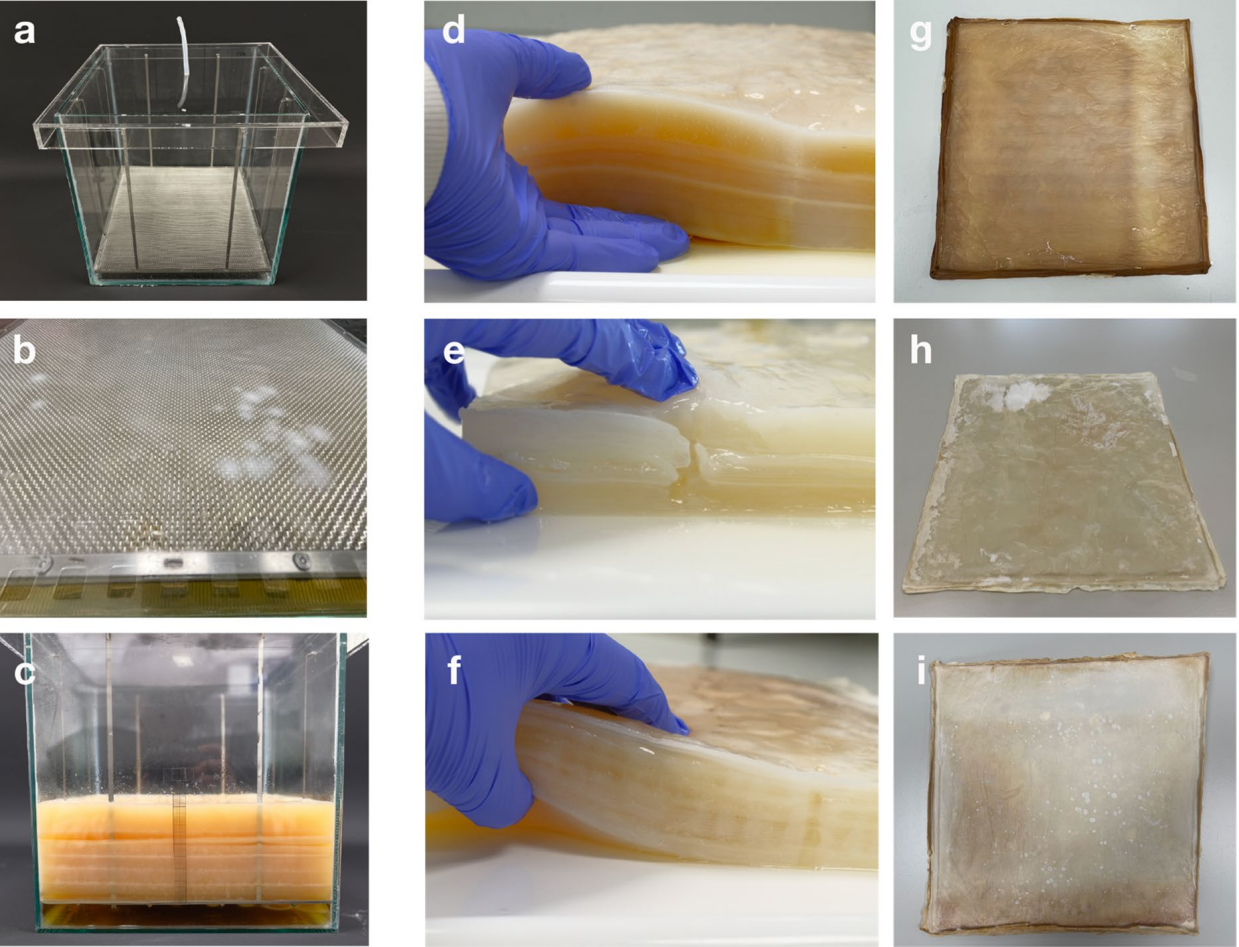
Large scale production of BC pellicles using MDV based bioreactor. **a** The 10 L growth vessel with supporting mesh scaffold and intermittent feeding tube; **b** Pellicle produced after 5 days of culturing with a patchy morphology; **c** Pellicle production on day 96; **d**, **g** Pellicles harvested at day 96 with the corresponding dried pellicle; **e**, **h** Pellicles harvested at day 66 with the corresponding dried pellicle; **f**, **i** Pellicle harvested at day 48 with the corresponding dried pellicle

**Fig. 8:**
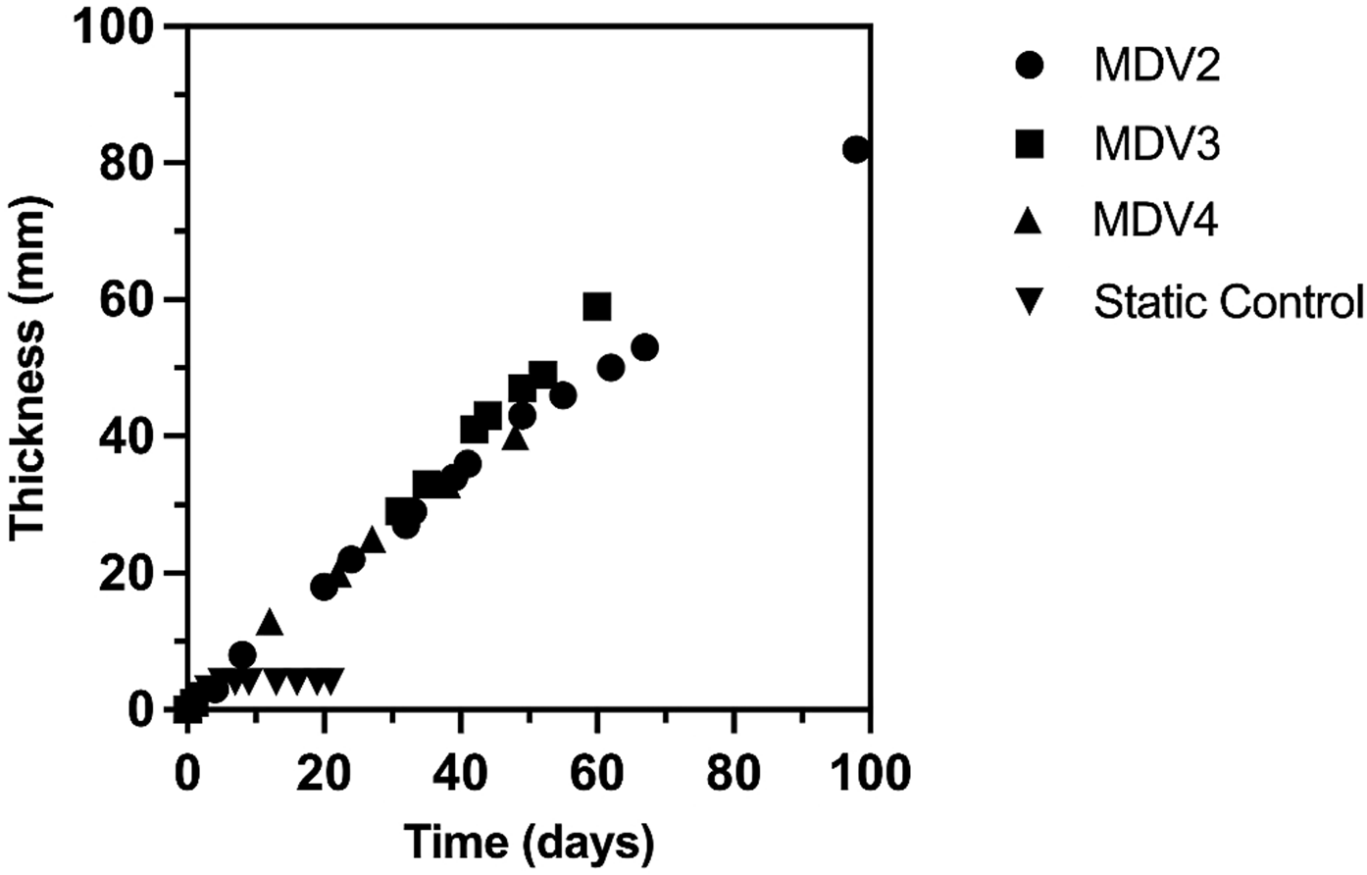
Growth of BC pellicles in upscaled cultures. MDV2 refers to the BC production from 96 days; MDV3 refers to the BC production from 66 days and MDV4 refers to the BC production from 48 days

To further test the tractability of the supportive mesh and intermittent feeding at a larger scale, cultures were upscaled to 10 L. In total, 3 volumes were tested, each was set up to achieve different pellicle thickness by culturing for different length of period: 96 days (MDV2), 66 days (MDV3), and 48 days (MDV4) (Fig. 7). As expected, cultures that were cultivated for longer produced BC that were thicker as there were more medium delivered by intermittent feeding (Fig. 8). This contrasted with the control (Fig. S1), which was grown in the same vessel through traditional static fermentation without mesh. Results here indicate that the supportive mesh and intermittent feeding does not increase the rate of production, but it prevents the stagnation in production (Fig. 8). When comparing the physical characteristics, BC produced with a supportive mesh and intermittent feeding had significantly increased yield, thickness, and dry weight (SI. Table S1).

Much like the smaller scale 800 mL beaker cultures, the mesh support anchors the growing pellicle and prevents submersion. Thus, the setup supports growth at the air–liquid interface. It was demonstrated that this setup could sustain cultivation for at least 98 days and was capable of producing a pellicle up to 82 mm thick, at which point it was deliberately stopped. Furthermore, to display control over the thickness of the pellicle, pellicles were also produced at 60 mm and 40 mm for 66 days and 48 days, respectively (Fig. 8 and SI. Table 1). The attribute of continuous production over time is retained. In contrast, the static control without mesh reached the stationary phase at day 5 and maintained the same thickness for 16 more days. After the BC were harvested, the yield was measured in terms of pellicle weight, as shown in SI. Table 1. Once dried, all pellicles produced by the bioreactor experience a 99% reduction in both thickness and weight.

As shown in these results, the advantage of continual and indefinite growth of BC is the ability to control the wet thickness of the product. Unlike existing bioreactor types for producing BC, such as airlift (Pa’e, 2009) and rotating disc (Lin et al. 2014)), which produce BC with heterogenous properties and lacking control, the BC pellicles produced in the MDV exhibit homogeneous properties throughout the 3D volume.

### SEM morphological analysis for BC produced in MDV based bioreactor

**Fig. 9:**
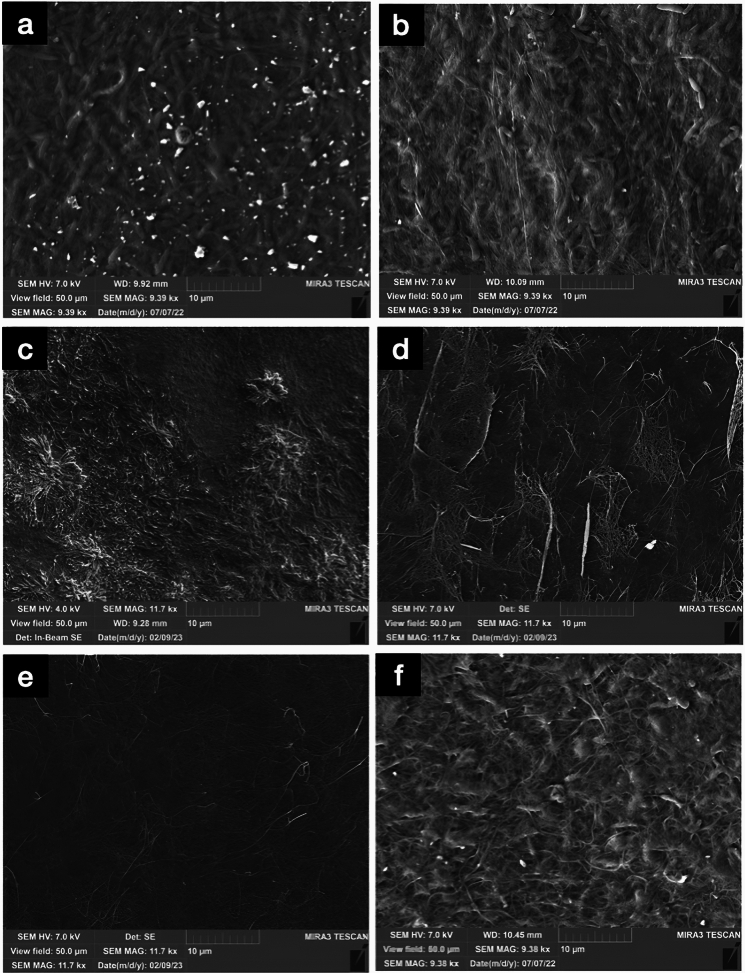
Morphology analysis for BC pellicles produced **a** from 800 MDV based bioreactor; **b** produced from conventionally static cultures without mesh; **c** from 10 L MDV based bioreactor (top surface); **d** from 10 L MDV based bioreactor (bottom surface); **e** from 10 L conventional static conditions wihtout mesh (top surface); **f** from 10 L conventional static conditions without mesh (bottom surface)

The BC pellicles produced from 800 mL cultures were observed under SEM (Fig. 9a and 9b). The micrographs showed no significant difference in morphology for both BC samples grown under conventionally static methods without mesh or in MDV bioreactor. With BC samples from the 10 L cultures, it was observed that there were clear morphological differences in the top and bottom surface of the pellicle. The top surface exhibited to have numerous crystal-like protrusions that were part of the cellulose fibre network (Fig. 9c). In contrast, the bottom side, which was anchored to the mesh, did not have these crystal-like structures and it was observed that the bottom side had a looser cellulosic network (Fig. 9d). We hypothesize that looser cellulosic network may be associated with the harvesting process, particularly the act of detaching the pellicle from the mesh network. This observation is supported by the fact that such morphologies were hardly observed in BC pellicles from conventional static conditions. The crystal-like morphologies were assumed to be nascent cellulosic fibres that were growing towards the direction of higher oxygen tension (i.e. the Z-axis). With BC samples produced by the conventionally static method without mesh (Fig. 9e, f), the crystal protrusions were not observed on the top surface, and the surface area exhibited a smooth cellulosic network. In contrast, the bottom surface of the cellulose exhibits a complex structure, including visible bacterial cells, a feature that was not present in BC produced by the MDV bioreactor. It is plausible that under these conditions, nutrients accumulate beneath the pellicle, leading bacterial cells to remain and proliferate on the bottom surface where nutrients are more accessible. Conversely, in the MDV system, the fresh medium is introduced to the top layer of the pellicle, causing the cells to proliferate upwards due to chemotaxis. Furthermore, as the pellicle thickens, the oxygen level beneath it significantly decreases in the conventional static reactor. This reduction in oxygen could also contribute to the decreased cell population at the bottom of the pellicle.

### Glucose consumption of BC cultures

**Fig. 10:**
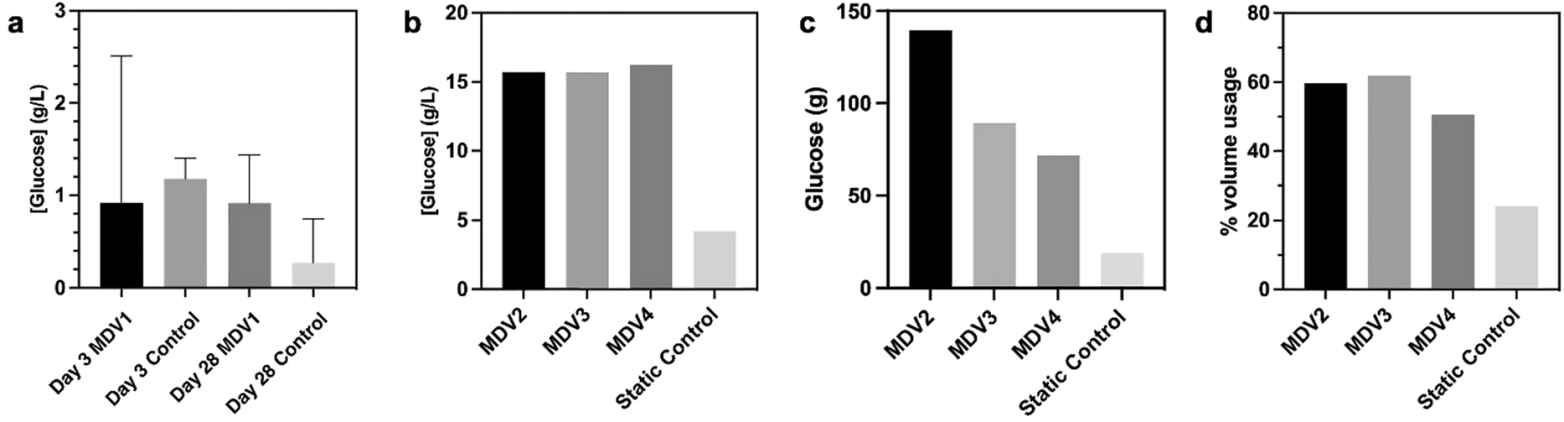
Glucose consumption **a** of 800 mL cultures at day 3 and day 28; **b** of cultures from 10 L bioreactors; **c** Conversion of glucose to BC from 10 L bioreactors; **d** The volume efficiency of BC pellicle produced against the volume of medium used. Error bars indicate standard error of mean and n = 3

To further investigate the effect of the MDV system, glucose consumption was also investigated. Growth medium was collected at the start and end of 800 mL cultures. There was no significant difference observed in overall glucose consumption at the harvest point (Fig. 10a). In both samples, the total amount of glucose was exhausted, which suggests complete usage of glucose. This indicates that both supported and unsupported cultures utilised the same amount of glucose while MDV based bioreactor produced more BC.

The trend of complete utilisation of glucose was not observed in upscaled cultures in MDV based bioreactor (Fig. 10b). Regardless of cultivation time or final thickness, each culture maintained a glucose concentration of around 16 gL^−1^. The relatively high amount of leftover glucose supports the hypothesis that there are more active bacterial cells at the surface of the pellicle, maybe through chemotaxis phenomenon in MDV bioreactor when the fresh medium was fed into the vessel. Once accounted for total glucose added to the system, remaining glucose after harvest and BC yield, pellicles produced from MDV bioreactor on average have a total glucose conversion to BC of 46.90% (Fig. 10c). This is in contrast with conventional static cultures without mesh, which achieve a total glucose conversion to BC of 14.04%. Therefore, with the culmination of the method used here results in 3.3-fold better usage of glucose when compared to conventional static cultures without mesh. When accounting for water usage, it was found that the method presented here increases water usage efficiency. At a 10 L scale, cultures in the MDV bioreactor exhibited a volume efficiency of 57.34%, indicating the volume of wet pellicle produced relative to the total volume of medium utilised for supporting BC production. In contrast, conventional static cultures without mesh showed a volume efficiency of 24.00%. Therefore, the MDV system can enhance water usage efficiency by 2.4-fold (Fig. 10d).

These results suggest that interval feeding and mesh support in the MDV bioreactor result in better utilisation of glucose to produce BC. For the cellulose-producing bacteria, glucose is not only a substrate for cellulose production but also functions as a carbon source for energy generation, cellular growth, and the formation of organic acid by-products. It has been observed previously that bacteria in the genus of *Komagataeibacter* readily convert glucose to organic acid by-products (Toda et al. 1997). When glucose is used as a substrate, the production of acids lowers the pH of the culture. However, this conversion of glucose to acids directly competes with BC production, with as much as 40.03% of total glucose were observed to be converted to gluconic acids and, in contrast, only 19.05% of total glucose was converted to BC (Zhong et al. 2013). In the smaller scale MDV system, higher productivity of BC production was observed when compared to conventional static cultures with the same amount of glucose. By delivering the glucose to the most active region for BC production, it is possible that the cells are in an ideal condition for BC production and readily convert the glucose to cellulose rather than converting glucose to gluconic acids, such as once BC production reaches the stationary phase in static conventional cultures. Another factor worth considering for continual BC production is oxygen tension (Hornung et al. 2007). In certain strains of cellulose-producing species, slight hypoxic conditions have been reported to favour BC production, leading to a higher BC yield (Liu et al. 2019) and inversely, condition of hyperoxia can result in lower BC yield (Huang et al. 2022). It has also been suggested that direct feeding results in the dilution of enriched acidic by-products, which can lead to suboptimal pH conditions for BC production (Hornung et al. 2007). However, in the 10 L cultures, there was a reversal in the trend toward complete glucose utilization. It is presumed that this is due to hindered glucose utilization, potentially caused by the diffusion barrier posed by the bulk of the pellicle. This diffusion barrier may also affect oxygen access in the region beneath the pellicle. It can be inferred that without sufficient oxygen tension, the viability of aerobic *K. xylinus* may decrease, consequently reducing the metabolic consumption of glucose in that area. Further study would be needed to directly measure diffused oxygen levels in the medium as it would be interesting to overcome the low O_2_ environment to increase BC production.

### Mechanical properties of BC pellicles produced in MDV based bioreactor

**Fig. 11:**
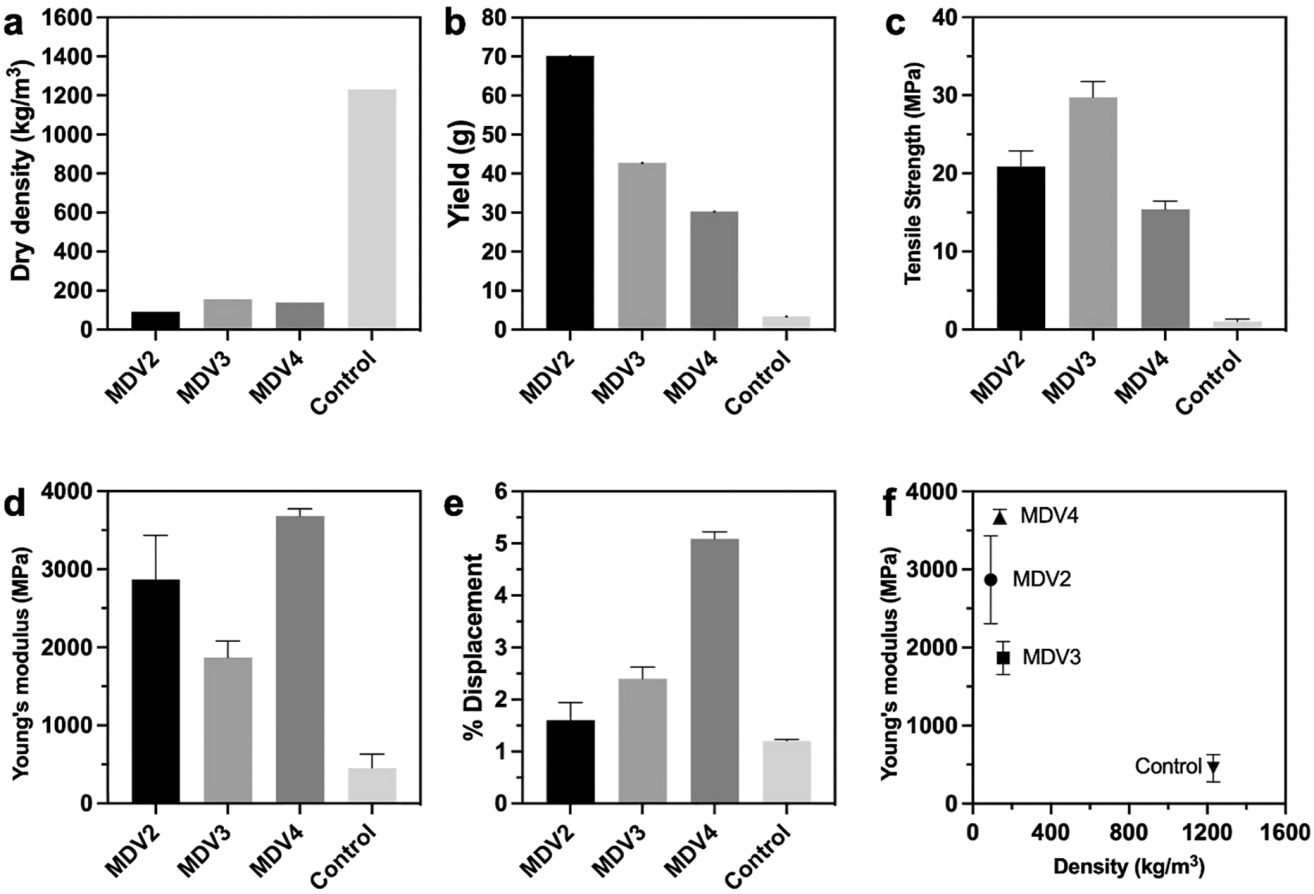
Material characteristics of BC pellicle produced from 10 L MDV based bioreactors; **a** dry density; **b** yield; **c** tensile strength; **d** Young’s modulus; **e** displacement; **f** Young’s modulus compared with density of BC. Error bars on c, d, e, and f indicate standard error of mean and n = 3

The material testing (Fig. 11) through tensile strength analysis revealed significant distinctions between samples grown in MDV system and conventional bioreactors. The mesh-supported samples exhibited higher elongation capability compared to the control group, even at increased thickness. However, they also displayed increased brittleness, with a notably higher Young's modulus than the BC produced from control cultures. Notably, the relationship between the thickness of the dry pellicle and Young's modulus was not linear. Unsurprisingly, there is a linear relationship between duration of growth and yield, as this relationship was suggested by Fig. 11b.

Additionally, the dry density of these samples was significantly lower than the control sample, with the thickest pellicle displaying a remarkably low density of 91.5 kg/m^3^. However, the differing thicknesses between MDV2-4 did not result in any significant change in dry density. It is likely that the intermittent feeding introduces interlayer spacing which causes a looser network between layers and therefore a lower density material. It is possible that the density would decrease if the volume of media used for intermittent feeding were increased, but this is not tested here.

Furthermore, the samples from MDV system demonstrated superior strength, with a tensile strength of 15–30 MPa compared to the control's 1 MPa. Although this data did not correlate with thickness, it did align with the trend observed in Young's modulus, suggesting a potential link between increased strength and brittleness. Additionally, the dry density of these samples showed a negative correlation with thickness, with the thickest pellicle displaying a remarkably low density of 91.5 kg/m^3^ compared to 156 kg/m^3^ for the thinnest. The BC produced from the MDV system exhibits expanded properties, featuring lower density and higher Young's Modulus, making it promising for lightweight applications in areas such as aerospace structures, automotive components, high-performance sports equipment, and similar fields.

### Advantage of supportive mesh and intermittent feeding in MDV bioreactor

This BC fermentation method from the MDV bioreactor represents an advancement over the previously described direct feeding bioreactor, which utilised aerosolised growth medium (Hornung et al. 2007) and intermittent feeding (Hsieh et al. 2016). Like similar studies, BC production here is continuous and potentially indefinite, facilitated by directly supplying nutrients to the air liquid interface thereby supporting further BC growth. In this system, akin to the aerosol bioreactor, nutrient diffusion barriers are effectively eliminated, as nutrients are delivered directly to the actively growing area rather than through diffusion from beneath the pellicle in conventional static culture. The MDV bioreactor with its supporting mesh scaffold and intermittent feeding offers significant advantages in fermentation efficiency, particularly glucose usage and water consumption. In addition, the method allows for control over the growth rate of the BC pellicle (measured in mm per day) and reduces contamination risks associated with aerosolized mediums. The work here demonstrates a new method to optimising yield and cost-effectiveness independent of nutrient composition in culture medium or modifications to the cellulose-producing organisms (e.g. strains and genetic engineering). Therefore, it would be interesting to combine the method explored here with optimal growth media (Abdelraof et al. 2019) and high yield cellulose producers (Florea et al. 2016) to further enhance cellulose production yields. An interesting aspect to this method of culturing, as it produces the pellicle layer-by-layer in a controlled manner, is the potential ability to introduce changes at precise and specific layers of cellulose. Functional changes can be hypothetically introduced to specific layers of the pellicle by the addition of additives. These additives can take the form of carbon sources (Żywicka et al. 2018), embedment of proteins (Gilmour et al. 2023), introduction of enzymatic reactions (Gilbert et al. 2021) or even embedment of partner species like *Saccharomyces cerevisiae* (Gilbert et al. 2021). This has the potential of introducing controllable functional changes across the bulk of the material and may have the potential of directing functional changes in 2D space (across the x and y-axis of the pellicle) and eventually 3D space (across the z- axis of the pellicle).

## Conclusion

In the MDV bioreactor, utilising a mesh scaffold and intermittent feeding enables precise control over the thickness of the produced BC pellicle while minimizing the formation of separate layers. Intermittent feeding introduces interlayer spacing, which causes a looser network between layers and therefore connected laminations. This is, however, quite different from the separate multilayering that occurs in intermittent feeding setups without the mesh support. The scaffold support ensures that growing BC pellicles remain at the air–liquid interface, exposing the most actively growing layer to nutrients introduced during intermittent feeding. This approach facilitates the production of thicker BC pellicles, surpassing those produced by conventional static methods. The combination of a supportive mesh and intermittent feeding proves adaptable at various scales, allowing for continuous and indefinite growth at a rate of nearly 1 mm in thickness per day. Furthermore, this method has resulted in a 3.4-fold increase in glucose conversion and a 2.4-fold increase in water efficiency. With the novel properties produced from the MDV bioreactor, the potential application fields of BC materials could expand, enabling adaptability for industrial use.

## Supplementary Information

Below is the link to the electronic supplementary material.

Supplementary file1 (DOCX 902 KB)

Supplementary file2 (DOCX 321 KB)

## Data Availability

No datasets were generated or analysed during the current study.

## References

[CR1] Chao YP, Sugano Y, Kouda T et al (1997) Production of bacterial cellulose by Acetobacter xylinum with an air-lift reactor. Biotechnol Tech 11:829–832. 10.1023/A:1018433526709/METRICS

[CR2] Cheng H-P, Wang P-M, Chen J-W, Wu W-T (2002) Cultivation of Acetobacter xylinum for bacterial cellulose production in a modified airlift reactor. Biotechnol Appl Biochem 35:125–132. 10.1111/J.1470-8744.2002.TB01180.X 11916454 10.1042/ba20010066

[CR30] Cheng Q, Wang S, Rials TG (2009) Poly(vinyl alcohol) nanocomposites reinforced with cellulose fibrils isolated by high intensity ultrasonication. Compos Part A Appl Sci Manuf 40:218–224. 10.1016/j.compositesa.2008.11.009

[CR31] Czaja W, Krystynowicz A, Bielecki S, Brown RM (2006) Microbial cellulose - The natural power to heal wounds. Biomaterials 27:145–151. 10.1016/j.biomaterials.2005.07.035 16099034 10.1016/j.biomaterials.2005.07.035

[CR3] Dade-Robertson M, Arnardottir T, Gilmour K et al (2023) Engineered living fabrication. Combining hardware, wetware and software for the non-entropic guided growth of microbial cellulose. In: Proceedings of the 43rd annual conference of the association for computer aided design in architecture, Denver, US, 21-28 October 2023, pp 94–207

[CR4] Faina A, Nejati B, Stoy K (2020) Evobot: an open-source, modular, liquid handling robot for scientific experiments. Appl Sci 10:814

[CR34] Florea M, Hagemann H, Santosa G et al (2016) Engineering control of bacterial cellulose production using a genetic toolkit and a new cellulose-producing strain. Proc Natl Acad Sci USA 113. 10.1073/pnas.1522985113 10.1073/pnas.1522985113 PMC4914174 27247386

[CR35] Gilbert C, Tang T, Ott W et al (2021) Living materials with programmable functionalities grown from engineered microbial co-cultures. Nat Mater. 10.1038/s41563-020-00857-5 33432140 10.1038/s41563-020-00857-5

[CR36] Gilmour KA, Aljannat M, Markwell C et al (2023) Biofilm inspired fabrication of functional bacterial cellulose through ex-situ and in-situ approaches. Carbohydr Polym 304:120482. 10.1016/j.carbpol.2022.120482 36641190 10.1016/j.carbpol.2022.120482

[CR5] Ho Jin Y, Lee T, Kim JR et al (2019) Improved production of bacterial cellulose from waste glycerol through investigation of inhibitory effects of crude glycerol-derived compounds by Gluconacetobacter xylinus. J Ind Eng Chem 75:158–163. 10.1016/J.JIEC.2019.03.017

[CR6] Hornung M, Ludwig M, Gerrard AM, Schmauder HP (2006) Optimizing the production of bacterial cellulose in surface culture: evaluation of substrate mass transfer influences on the bioreaction (Part 1). Eng Life Sci 6:537–545. 10.1002/ELSC.200620162

[CR7] Hornung M, Ludwig M, Schmauder HP (2007) Optimizing the production of bacterial cellulose in surface culture: a novel aerosol bioreactor working on a fed batch principle (Part 3). Eng Life Sci 7:35–41. 10.1002/ELSC.200620164

[CR8] Hsieh J-T, Wang M-J, Lai J-T, Liu H-S (2016) A novel static cultivation of bacterial cellulose production by intermittent feeding strategy. J Taiwan Inst Chem Eng 63:46–51. 10.1016/j.jtice.2016.03.020

[CR9] Huang LH, Li XJ, Wang YT et al (2022) Enhancing bacterial cellulose production with hypoxia-inducible factors. Appl Microbiol Biotechnol 106:7099–7112. 10.1007/S00253-022-12192-7/FIGURES/7 36184690 10.1007/s00253-022-12192-7

[CR10] Hur DH, Rhee HS, Lee JH et al (2020) Enhanced production of cellulose in Komagataeibacter xylinus by preventing insertion of IS element into cellulose synthesis gene. Biochem Eng J 156:107527. 10.1016/J.BEJ.2020.107527

[CR11] Kamiński K, Jarosz M, Grudzień J et al (2020) Hydrogel bacterial cellulose: a path to improved materials for new eco-friendly textiles. Cellulose 27:5353–5365. 10.1007/S10570-020-03128-3

[CR39] Klemm D, Schumann D, Udhardt U, Marsch S (2001) Bacterial synthesized cellulose - Artificial blood vessels for microsurgery. Prog Polym Sci (Oxford) 26:1561–1603. 10.1016/S0079-6700(01)00021-1

[CR38] Klemm D, Heublein B, Fink H, Bohn A (2005) Cellulose: fascinating biopolymer and sustainable raw material. Angew Chem Int Ed 44:3358–3393. 10.1002/anie.200460587 10.1002/anie.200460587 15861454

[CR12] Krystynowicz A, Czaja W, Wiktorowska-Jezierska A et al (2002) Factors affecting the yield and properties of bacterial cellulose. J Ind Microbiol Biotechnol 29:189–195. 10.1038/SJ.JIM.7000303 12355318 10.1038/sj.jim.7000303

[CR13] Lin SP, Hsieh SC, Chen KI et al (2014) Semi-continuous bacterial cellulose production in a rotating disk bioreactor and its materials properties analysis. Cellulose 21:835–844. 10.1007/S10570-013-0136-8/FIGURES/7

[CR14] Liu C, Zhang W, Song S, Li H (2019) A novel method to improve carboxymethyl cellulose performance in the flotation of talc. Miner Eng 131:23–27. 10.1016/J.MINENG.2018.11.003

[CR15] Lv P, Zhou H, Mensah A et al (2018) A highly flexible self-powered biosensor for glucose detection by epitaxial deposition of gold nanoparticles on conductive bacterial cellulose. Chem Eng J 351:177–188. 10.1016/J.CEJ.2018.06.098

[CR16] Padrão J, Gonçalves S, Silva JP et al (2016) Bacterial cellulose-lactoferrin as an antimicrobial edible packaging. Food Hydrocoll 58:126–140. 10.1016/J.FOODHYD.2016.02.019

[CR40] Pa’E NB (2009) Rotary Disk Reactor for Enhanced Production of Microbial Cellulose. Masters Thesis, Universiti Teknologi Malaysia

[CR17] Sani A, Dahman Y (2010) Improvements in the production of bacterial synthesized biocellulose nanofibres using different culture methods. J Chem Technol Biotechnol 85:151–164. 10.1002/JCTB.2300

[CR50] Song H-J, Li H, Seo J-H et al (2009) Pilot-scale production of bacterial cellulose by a spherical type bubble column bioreactor using saccharified food wastes. Korean J Chem Eng 26:141–146. 10.1007/s11814-009-0022-0

[CR18] Toda K, Asakura T, Fukaya M et al (1997) Cellulose production by acetic acid-resistant Acetobacter xylinum. J Ferment Bioeng 84:228–231. 10.1016/S0922-338X(97)82059-4

[CR19] Wang Z, YuyuLi EJ et al (2023) Sustainable bacterial cellulose-based composite aerogels with excellent flame retardant and heat insulation. Cellulose 30:9563–9574. 10.1007/S10570-023-05461-9/FIGURES/8

[CR20] Zeng M, Laromaine A, Feng W et al (2014) Origami magnetic cellulose: controlled magnetic fraction and patterning of flexible bacterial cellulose. J Mater Chem C Mater 2:6312–6318. 10.1039/C4TC00787E

[CR21] Zhang Y, Chen Y, Li X et al (2021) Bacterial cellulose hydrogel: a promising electrolyte for flexible zinc-air batteries. J Power Sources 482:228963. 10.1016/J.JPOWSOUR.2020.228963

[CR22] Zhong C, Zhang GC, Liu M et al (2013) Metabolic flux analysis of Gluconacetobacter xylinus for bacterial cellulose production. Appl Microbiol Biotechnol 97:6189–6199. 10.1007/S00253-013-4908-8/FIGURES/7 23640364 10.1007/s00253-013-4908-8

[CR23] Żywicka A, Junka AF, Szymczyk P et al (2018) Bacterial cellulose yield increased over 500% by supplementation of medium with vegetable oil. Carbohydr Polym 199:294–303. 10.1016/J.CARBPOL.2018.06.126 30143132 10.1016/j.carbpol.2018.06.126

