# Supplementary material for: Enhanced production of bacterial cellulose with a mesh dispenser vessel-based bioreactor
Source: Cellulose (Lond). 2025 Jan 29;32(4):2209–26. doi: 10.1007/s10570-024-06367-w (PMC11933169; doi:10.1007/s10570-024-06367-w)
Supplement: Supplementary file 1 — Supplementary file1 (DOCX 902 KB) [file 10570_2024_6367_MOESM1_ESM.docx]

**Supplementary Information**

**
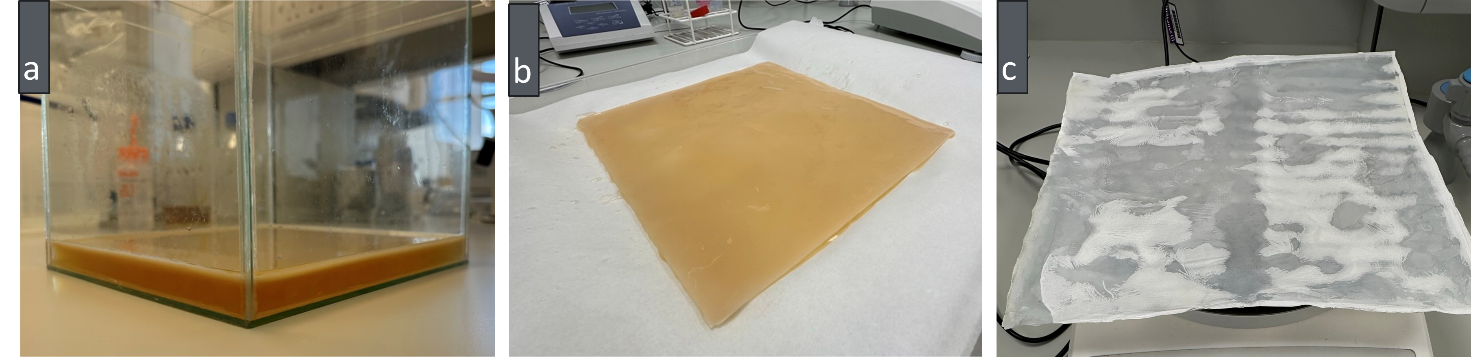
Fig S1**. BC production in 10 L glass vessel through traditional static fermentation. (a) BC formation feeding after 21 days of culturing; (b) Harvested BC pellicles; (c) Dried BC pellicle

**Table 1.** Culturing time and harvested BC physical characteristics obtained from MDV2, 3, and 4.

|  | **Culturing time (days)** | **Wet thickness (mm)** | **Wet weight (g)** | **Dry thickness (mm)** | **Dry weight/Yield (g)** | **Dry density (kg/m3)** |  |
| --- | --- | --- | --- | --- | --- | --- | --- |
|  |  |  |  |  |  |  |  |
| MDV2 | 96 | 80 | 4929.12 | 0.14 | 70.16 | 91.55 |  |
| MDV3 | 66 | 60 | 2319.32 | 0.05 | 42.73 | 156.12 |  |
| MDV4 | 48 | 40 | 2599.00 | 0.04 | 30.26 | 138.2 |  |
| Control | 21 | 5 | 322.81 | 0.0005 | 3.37 | 1231.28 |  |
