# Supplementary material for: Enhanced production of bacterial cellulose with a mesh dispenser vessel-based bioreactor
Source: Cellulose (Lond). 2025 Jan 29;32(4):2209–26. doi: 10.1007/s10570-024-06367-w (PMC11933169; doi:10.1007/s10570-024-06367-w)
Supplement: Supplementary file 2 — Supplementary file2 (DOCX 321 KB) [file 10570_2024_6367_MOESM2_ESM.docx]

**Supplementary material and data for Enhanced production of bacterial cellulose with a mesh dispenser vessel-based bioreactor**

| 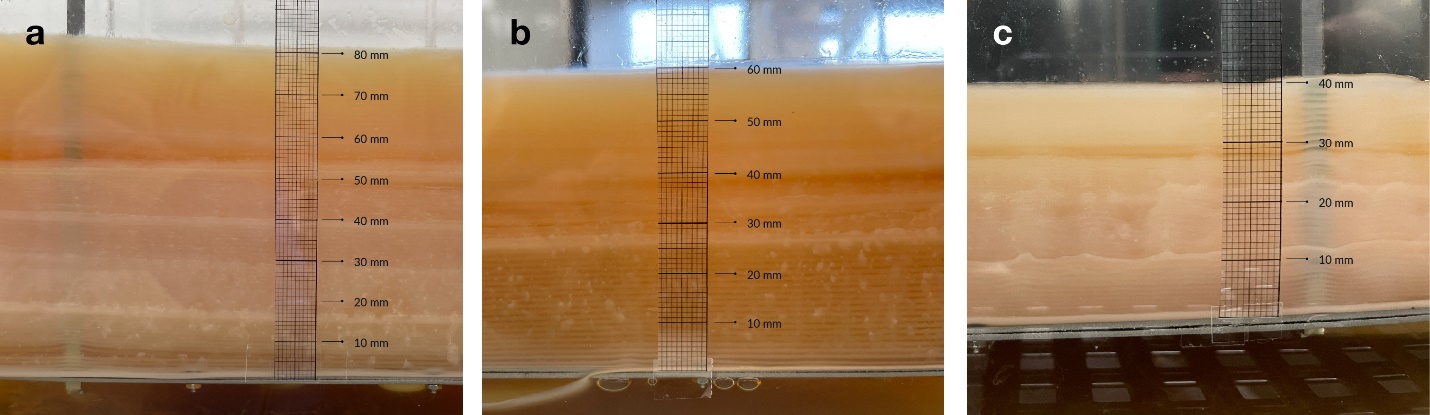 |
| --- |
| **Figure. S1** showing total thickness increase for each of the MDV methods in the bioreactor. Each line of the scale bar represents 1mm. (a) MDV2 shown with a growth amounting to 80 mm in thickness, (b) MDV3 with 60 mm thickness and, (c) MDV4 with 40 mm of BC growth thickness. |

  
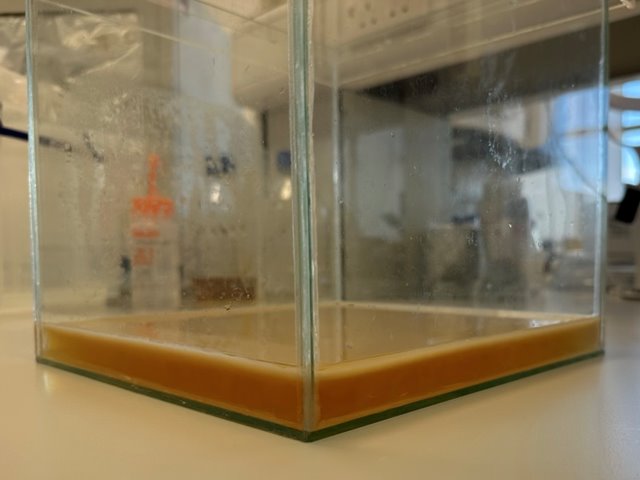

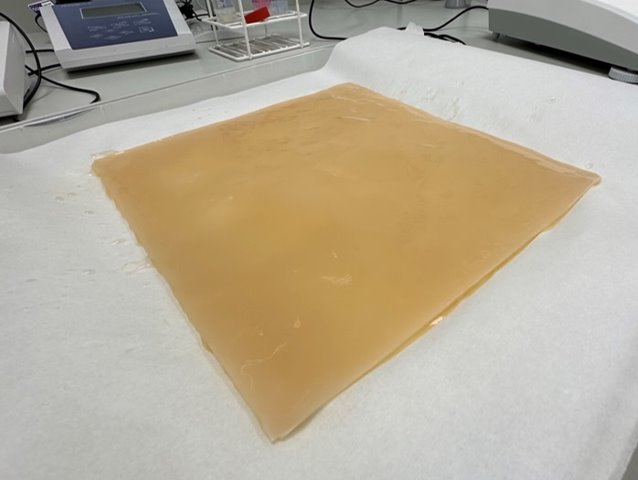

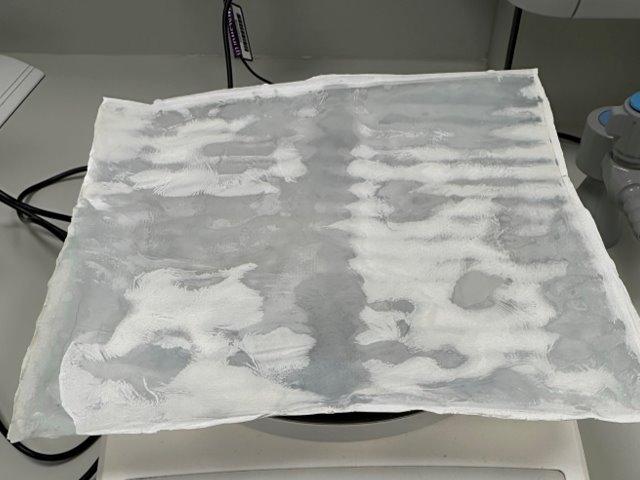


C

B

A

**Figure S2.** BC production in 10 L glass vessel through traditional static fermentation. (a) BC formation feeding after 21 days of culturing; (b) Harvested BC pellicles; (c) Dried BC pellicle​


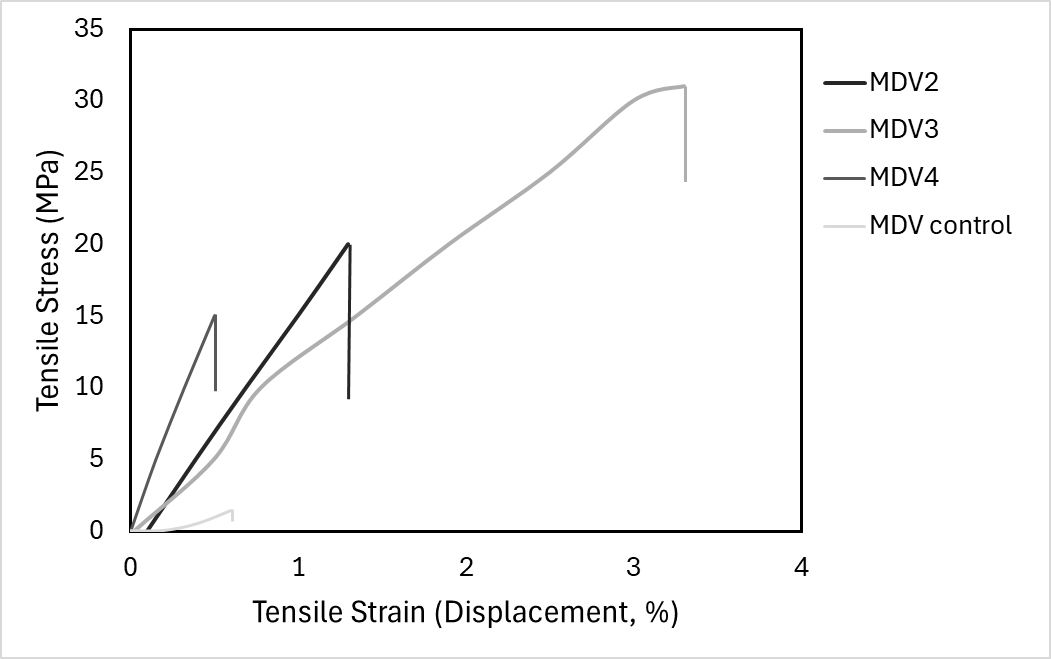


**Figure S3.** Typical Stress-Strain curves of pellicles from 10 L MDV bioreactor with intermittent feeding during tensile strength testing.
